# The potential predictive value and relationship of blood-based inflammatory markers with the clinical symptoms of Han Chinese patients with first-episode adolescent-onset schizophrenia

**DOI:** 10.3389/fpsyt.2024.1431350

**Published:** 2024-09-03

**Authors:** Zhihua Liu, Dali Lv, Jianfeng Li, Fuwei Li, Yanhua Zhang, Yongjie Liu, Chao Gao, Yafeng Qiu, Jun Ma, Ruiling Zhang

**Affiliations:** ^1^ Department of Psychiatry, The Fourth People’s Hospital of Nanyang, Nanyang, Henan, China; ^2^ Department of Psychiatry, The Fifth People’s Hospital of Luoyang, Luoyang, Henan, China; ^3^ Department of Psychiatry, Wuhan Mental Health Center, Wuhan, Hubei, China; ^4^ Department of Psychiatry, The Second Affiliated Hospital of Xinxiang Medical University, Xinxiang, Henan, China

**Keywords:** adolescent-onset schizophrenia, Han Chinese, inflammatory markers, predictive value, clinical symptoms

## Abstract

**Background:**

Inflammation is associated with the pathophysiology of schizophrenia. The blood markers for systemic inflammation include neutrophil-lymphocyte ratio (NLR), systemic immune-inflammation index (SII), lymphocyte-monocyte ratio (LMR), system inflammation response index (SIRI), and platelet-lymphocyte ratio (PLR). However, these inflammation markers and their relationships with clinical phenotypes among Han Chinese patients with first-episode adolescent-onset schizophrenia (AOS) is unclear. This investigation aimed to elucidate the impact of inflammation on Han Chinese AOS patients as well as the association of blood-based inflammation markers with clinical symptoms.

**Methods:**

Altogether, 203 Han Chinese individuals participated in this study, 102 first-episode AOS patients and 101 healthy controls. The assessment of inflammatory indices was based on complete blood cell count. Furthermore, schizophrenia-related clinical symptoms were evaluated using the five-factor model of the Positive and Negative Syndrome Scale (PANSS).

**Results:**

In Han Chinese first-episode AOS patients, levels of SIRI, PLR, SII, and NLR were significantly increased (*p < 0.001*), while LMR decreased (*p < 0.001*) compared to healthy controls. Furthermore, multivariate logistic regression showed that LMR, NLR, SII, and SIRI (all p < 0.05) were independently associated with AOS. Moreover, Receiver operating characteristics assessment indicated that NLR, SIRI, LMR, and SII could effectively distinguish AOS patients from healthy controls. Their areas under the curves were 0.734, 0.701, 0.715, and 0.730 (all *p < 0.001*). In addition, Correlation analysis revealed that LMR was negatively correlated with the PANSS total, negative, and cognitive factor scores (all *p < 0.05*); NLR was positively correlated with the cognitive factor score (*p < 0.01*); SII was negatively correlated with the positive factor score and positively with the negative and cognitive factor scores (all *p < 0.05*); SIRI was positively correlated with the PANSS total and cognitive factor scores (all *p < 0.01*).

**Conclusions:**

This research established the involvement of peripheral blood inflammatory markers (LMR, NLR, SII, and SIRI) with the clinical manifestations and pathophysiology of schizophrenia, and these can serve as screening tools or potential indices of the inflammatory state and AOS symptoms severity.

## Introduction

Schizophrenia is a chronic, disabling disease with complex etiology and highly heterogeneous clinical manifestations, and the search for the underlying causes and pathogenesis of the disease, as well as the pursuit of effective treatment, has always been a hot issue in psychiatry (1, 2). Furthermore, it has been observed that schizophrenia’s peak onset is in the adolescent and early adult stages (3, 4). The schizophrenia originating in the adolescent period (13 - 18 years old) is called adolescent-onset schizophrenia (AOS) (5, 6). AOS is observed 5 times less often than adult-type schizophrenia, with an incidence of 0.5 - 1% (6). Since AOS occurs in the critical period of neural development, it usually has an asymptomatic onset, prolonged course, severe condition, and poor long-term prognosis (3, 7). Objective biological markers for schizophrenia are lacking, and its etiologies, as well as pathogenesis, have not been comprehensively studied yet (8, 9). Therefore, the identification of effective biological markers and their relationships with schizophrenia-related clinical symptoms is significant for the further understanding of its etiologies and pathophysiological mechanisms, as well as for its diagnosis and treatment, especially of AOS.

Recently, more and more evidence has shown that the aberrant immune system, including inflammatory responses, is closely related to the etiology and progression of schizophrenia (10). This inflammatory theory is supported by the increased pro-inflammatory cytokines (11, 12) [interleukins (IL)-6, IL-2, IL-8, TNF-α, IL-1, *etc.*], autoantibodies (13) (IgG, IgA, *etc.*), oxidative stress products (14), and maternal infection in the prenatal period (15) (influenza virus, toxoplasma, herpes simplex virus, measles virus, rubella virus, *etc.*). Furthermore, research on first-episode schizophrenia also indicated immune abnormalities in the patient’s peripheral blood, such as increased lymphocytes and inflammatory markers, including cytokines and C-reactive protein (CRP) (16, 17). Moreover, the association of inflammation with schizophrenia is also validated by the addition of non-steroidal anti-inflammatory drugs (NSAIDs) in antipsychotic drugs to reduce patient’s positive symptoms (18). However, clinical and laboratory studies have not found relatively stable inflammatory markers related to schizophrenia (19, 20).

Recently, relatively stable immune-inflammatory biomarkers have been identified, including monocyte-lymphocyte ratio (MLR), platelet-lymphocyte ratio (PLR), lymphocyte-monocyte ratio (LMR), and neutrophil-lymphocyte ratio (NLR) (21–23). Furthermore, Hu et al. proposed a novel inflammatory marker in 2014, the systemic immune-inflammatory index (SII) (24), and was initially used for the prognosis of hepatocellular carcinoma patients. SII is a biomarker that integrates platelet, neutrophil, and lymphocyte counts, and its calculation method is to multiply the platelet count by NLR. Moreover, it has been observed that SII can predict the severity and prognosis of some physical disorders, such as cardiovascular diseases, pancreatitis, and ischemic stroke (25–27). In addition, the systemic inflammatory response index (SIRI) is a new inflammation-based bio-index that is based on the peripheral blood levels of monocytes, neutrophils, and lymphocytes (28). It has been indicated that SIRI can serve as a prognostic factor for cancers, including nasopharyngeal carcinoma and cholangiocarcinoma, as well as stomach, esophageal, and pancreatic cancers (19). The SIRI is calculated by multiplying the monocyte count by NLR. Studies have found that SII and SIRI are significantly comprehensive inflammatory markers that can identify a wide range of systemic inflammatory reactions and better reflect the host’s inflammatory and immune response balance than MLR, PLR, and NLR (29, 30). Furthermore, it is noteworthy that these inflammatory indicators are assessed using the peripheral whole blood cell count, which is easy to obtain, low in price, and a potential biomarker.

Research has also indicated that compared with the healthy controls, the patients with schizophrenia (31, 32) and non-affective mental disorders (21) had a substantially higher NLR and MLR, while reduced NLR, which had a protective effect on schizophrenia (33). Furthermore, a study on patients with first-episode psychosis (34) also revealed increased NLR, PLR, and MLR. These findings further validate the association of inflammation with the occurrence and development of schizophrenia and that indicators including MLR, NLR, and PLR can potentially predict schizophrenia severity and prognosis, as well as monitor the therapeutic effect. Currently, there are only a few studies associating SII and SIRI with mental disorders (35). The aforementioned studies were all performed on adults, while research on first-episode schizophrenia is very limited, with inconsistent and contradictory results (34–37). Furthermore, research on AOS is even more rare. Due to the different cytokines produced in children, adolescents, and adults, the previous research results in the adult patient group may not apply to children and adolescents (38). Recently, a report on PLR, NLR, and MLR in adolescent schizophrenia was published; however, the included sample size was small (n = 30), it was not based on the first episode, and the factor of antipsychotic drugs was not excluded; therefore, it could not completely reflect the abnormality of inflammatory indicators caused by the disease itself (39). Moreover, there is no related research on Chinese Han adolescent patients. Overall, the association of inflammation with schizophrenia-related clinical symptoms is still not clear (40). Some research indicates a positive correlation of inflammatory markers with negative or positive symptoms, while others indicate negative or no correlations; therefore, the results are contradictory (40–43). Furthermore, only a few studies compare various inflammatory markers and their correlation with schizophrenia-related clinical symptoms, and studies on AOS are even more rare.

Compared with the adulthood onset of schizophrenia, AOS patients have a more severe neurobiological form with higher genetic susceptibility (44, 45), which causes severe clinical symptoms, prolongs the disease course, has a high recurrence rate, and poor prognosis and social function recovery, which requires an increased dose of antipsychotic drugs (46). Moreover, first-episode AOS patients are less affected by environmental factors (such as family and social stress) after adulthood; therefore, comprehensive research on AOS will indicate the underlying etiology and pathogenesis of schizophrenia (47).

This investigation aimed to elucidate how inflammation contributes to schizophrenia and the association of blood-based inflammation markers with symptom severity [assessed by Positive and Negative Syndrome Scale (PANSS)] in Han Chinese AOS patients. Based on the inflammatory theory and previous findings, it was hypothesized that (1) compared with the healthy controls, the Han Chinese AOS patients have increased PLR, SIRI, NLR, and SII, while reduced LMR; (2) PLR, SIRI, NLR, LMR, and SII are potent indices that can differentiate patients from healthy adolescents; (3) PLR, SIRI, NLR, LMR, and SII can indicate inflammation severity and is associated with first-episode AOS-related clinical symptoms. Understanding the association of blood inflammatory indices with clinical manifestations of AOS will provide valuable insights into schizophrenia pathogenesis and potentially identify novel treatment targets.

## Materials and methods

### Participants

This research included 102 Han Chinese first-episode AOS (aged 12 - 18 years) patients enrolled at the Nanyang Fourth People’s Hospital between January 2021 and December 2023. Furthermore, 101 healthy adolescents were randomly selected as controls during the same period from the outpatient medical record database of the Health Examination Center of the Fourth People’s Hospital of Nanyang City. These adolescents were from different communities in Nanyang City. The healthy controls and Han Chinese AOS patients were matched in terms of total education years, gender, ethnicity, and age.

Patients (1) who met the schizophrenia diagnostic criteria proposed in the 10^th^ edition of the International Classification of Diseases (ICD-10); (2) with first onset without medication; (3) with < 2 years of total disease course; (4) had Han nationality; (5) were aged between 12 - 18 years; (6) with the total education years ≥ 6 years; (7) with > 70 IQ were selected for this investigation.

Individuals (1) with a history of alcohol, tobacco, or drug abuse; (2) with mental retardation; (3) with intellectual disabilities; (4) who are deaf-mute; (5) with chronic physical illnesses, particularly those linked with systemic inflammatory responses such as fever, infection, acute or chronic endocrinological diseases were excluded from the study.

### Data collection

Fasting cubital vein blood was sampled from all the included participants. Sysmex XN-1000 (XN-1000, Sysmex, Japan) automatic blood cell analyzer was employed to assess complete blood cell count. Data and laboratory results were acquired from the electronic medical records system. Furthermore, PLR, SIRI, LMR, NLR, and SII values were assessed and compared between the two cohorts.

### Clinical evaluation

Characteristic symptoms of psychosis were assessed *via* the PANSS, which comprises 30 items (48); Further analyses were conducted using the five-factor model of the PANSS as proposed by Wallwork and colleagues (49) and the more recent guidance on assessment of negative symptoms in schizophrenia by the European Psychiatry Association (50). The five factors are: the positive factor (PANSS-PS, items P1, P3, P5, G9), the negative factor (50) (PANSS-NS, items N1, N2, N3, N4, N6), the cognitive/disorganized factor (PANSS-CS, items P2, N5, G11), the excited factor (PANSS-ES, items P4, P7, G8, G14), and the depressed factor (PANSS-DS, items G2, G3 and G6). The higher scores, the more severe symptoms. Two independent, well-trained psychiatrists assessed the PANSS, with the same psychiatrist per patient during treatment.

### Ethical considerations

This low-risk observational retrospective study has been submitted to the ethical board of the Fourth People’s Hospital of Nanyang City and was authorized by the institutional Ethics Committee. The requirement of informed consent was waived.

### Statistical analysis

SPSS 19.0 was employed to statistically analyze the acquired data. The data normality was assessed using the Kolmogorov-Smirnov test. Normally distributed data were depicted as mean ± standard deviation (mean ± SD), and for its intergroup comparison, an independent sample t-test was performed. Not normally distributed data were presented as median (interquartile range), and for its intergroup comparison, the Mann-Whitney U test was carried out. Furthermore, the independent variables linked with AOS were assessed *via* the multivariate logistic regression analysis. The statistically significant factors in the univariate analysis were utilized as independent variables, while the dependent variable was the occurrence of schizophrenia. Moreover, odds ratios (ORs) and 95% confidence intervals (CIs) were measured for the independent variables. In addition, the Han Chinese AOS patients and healthy controls differentiation efficiency of inflammatory markers was assessed *via* the receiver operating characteristic (ROC) curve. Pearson (for normally distributed variables) or Spearman (for not normally distributed variables) correlation tests were carried out to analyze the correlative relationship between peripheral inflammatory markers and PANSS scores. All statistical tests were two-tailed, and the statistical significance threshold was *p* < 0.05.

## Results

This research included 203 Han Chinese individuals; 102 AOS patients and 101 healthy controls. **Table 1** indicates the clinical and demographic characteristics of the included cohorts. No patient had a history of smoking and drinking. In the AOS group, there were 48 (47.1%) females and 54 (52.9%) males, with a mean age of 16 (15, 18) years. Whereas the healthy control cohort comprises 47 (46.5%) females and 54 (53.5%) males, with a mean age of 16 (15, 17) years. The two cohorts had no statistically significant variabilities in height, age, weight, gender, and body mass index (*p* > 0.05) (**Table 1**).

**Table 1:** Characteristics of patients with AOS and healthy comparison subjects.

Variable	Patients with AOS (n=102)	Healthy Controls (n=101)	t/z/χ^2^	*p*
**Age (years)**	16 (15,18)^a^	16 (15,17)^a^	1.076	0.283^b^
**Male (n%)**	54 (52.9%)	54 (53.5%)	0.006	0.940^c^
**Race**	Han Chinese	Han Chinese	–	–
**smoker**	no	no	–	–
**drinker**	no	no	–	–
**Family genetic history**	34 (33.3%)	–	–	–
**Disease duration (Months)**	2 (1,6)	–	–	–
**Weight (kg)**	58.27 ± 10.84^d^	60.51 ± 12.01^d^	-1.395	0.165^e^
**Height (m)**	1.66 ± 0.07	1.67 ± 0.08	-0.749	0.455
**BMI (kg/m2)**	21.13 ± 3.62	21.64 ± 3.24	-1.073	0.285
**PNASS-TS**	113.68 ± 8.37	–	–	–
**PANSS-PFS**	16.11 ± 2.67	–	–	–
**PANSS-NFS**	19.39 ± 3.72	–	–	–
**PANSS-CFS**	14.04 ± 1.66	–	–	–
**PANSS-EFS**	18.18 ± 2.98	–	–	–
**PANSS-DFS**	8.04 ± 1.96	–	–	–

AOS, Adolescent-onset Schizophrenia; BMI, Body mass index; PANSS, Positive and Negative Syndrome Scale; PNASS Total Score, PNASS-TS; PANSS-PFS, Positive Factor Score; PANSS-NFS, Negative Factor Score; PANSS-CFS, Cognitive Factor Score; PANSS-EFS, Excited Factor Score; PANSS-DFS, Depress Factor Score; ^a^Expressed as median (lower–upper quartiles); ^b^Mann–Whitney U test; ^c^Chi-square test; ^d^Expressed as mean ± standard deviation; ^e^Student’s t-test.

The results indicated that in AOS patients the levels of SIRI (*p <* 0.001), NLR (*p <* 0.001), white blood cell count (WBC) (*p* = 0.028), LMR (*p <* 0.001), PLR (*p <* 0.001), neutrophil count (*p <* 0.001), and SII (*p <* 0.001) were markedly enhanced than in healthy adolescents. Whereas, the healthy controls indicated notably reduced lymphocyte count (*p <* 0.001). No significant differences were observed in platelet count, mean platelet volume (MPV), and monocyte count between the two cohorts (all *p* > 0.05) (**Table 2**).

**Table 2:** Comparison of blood cell count parameters between the schizophrenia and healthy control groups.

Variables	Patients with AOS	Healthy Controls	t/z	*p*
**WBC (10^3^/uL)**	7.20 ± 2.18^a^	6.62 ± 1.45^a^	2.221	0.028^b*^
**Lymphocyte (10^3^/uL)**	1.93 ± 0.59	2.35 ± 0.55	-5.238	<0.001^*^
**Monocyte (10^3^/uL)**	0.45 ± 0.17	0.41 ± 0.17	1.590	0.113
**Neutrophil (10^3^/uL)**	4.72 ± 1.97	3.68 ± 1.15	4.596	<0.001^*^
**Platelet (10^3^/uL)**	279.88 ± 68.46	270.41 ± 53.39	1.100	0.273
**MPV (fl)**	9.96 ± 1.15	10.03 ± 2.15	-0.961	0.338
**LMR**	4.74 ± 1.86	6.27 ± 2.15	-5.421	<0.001^*^
**NLR**	2.22(1.45,3.56)^c^	1.55(1.20,2.05)^c^	-5.762	<0.001^d*^
**PLR**	157.55 ± 57.02	120.94 ± 32.99	5.606	<0.001^*^
**SII**	763.19 ± 465.11	438.30 ± 171.04	6.617	<0.001^*^
**SIRI**	0.91 (0.57,1.85)	0.56(0.41,0.85)	-5.656	<0.001^*^

WBC, White blood cell count; MPV, Mean platelet volume; LMR, Lymphocyte-monocyte ratio; NLR, Neutrophil–lymphocyte ratio; PLR, Platelet-lymphocyte ratio; SII, systemic immune-inflammation index; SIRI, system inflammation response index; ^a^Expressed as mean ± standard deviation; ^b^Student’s t-test; ^c^Expressed as median (lower–upper quartiles); ^d^Mann–Whitney U test; * indicates statistical significance.

The multivariate logistic regression results indicated that LMR (OR: 0.832, 95% CI: 0.694 - 0.998, *p* = 0.048), NLR (OR: 2.413, 95% CI: 1.392 - 4.184, *p* = 0.002), SII (OR: 1.002, 95% CI: 1.001 - 1.004, *p* = 0.006) and SIRI (OR: 2.853, 95% CI: 1.598 - 5.094, *p <* 0.001) were independently linked with AOS patients (**Table 3**).

**Table 3:** Multivariate logistic regression model for the association between AOS and inflammatory marker.

Variables	OR (95%CI)	*p*
**LMR**	0.832 (0.694-0.998)	0.048^*^
**NLR**	2.413 (1.392-4.184)	0.002^*^
**PLR**	1.005 (0.995-1.015)	0.315
**SII**	1.002 (1.001-1.004)	0.006^*^
**SIRI**	2.853 (1.598-5.094)	<0.001^*^

LMR, Lymphocyte-monocyte ratio; NLR, Neutrophil–lymphocyte ratio; PLR, Platelet-lymphocyte ratio; SII, systemic immune-inflammation index; SIRI, system inflammation response index; *indicates statistical significance.

**Figure 1:**
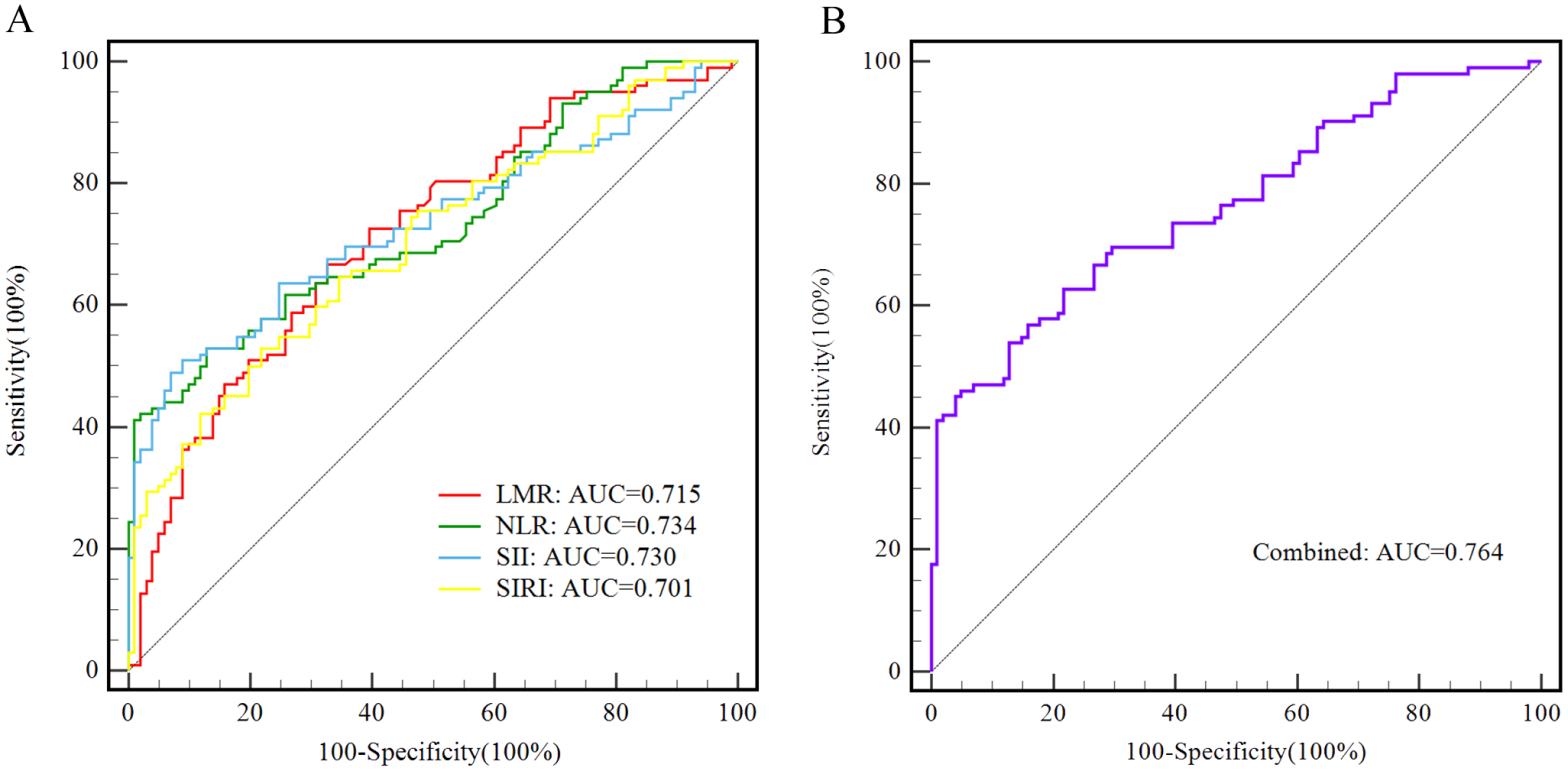
The ROC curves of LMR, NLR,SII and SIRI alone and in combined for differentiating adolescents with schizophrenia from healthy control group. **(A)** LMR is presented with a red line, with an AUC of 71.5%, a sensitivity of 66.67%, and a specificity of 67.33%; NLR is presented with a green line, with an AUC of 73.4%, a sensitivity of 41.18%, and a specificity of 99.01%; SII is presented with a blue line, with an AUC of 73.0%, a sensitivity of 49.02%, and a specificity of 93.07%; SIRI is presented with a yellow line, with an AUC of 70.1%, a sensitivity of 52.94%, and a specificity of 78.22%. **(B)** The combined model is presented with a purple line, with an AUC of 76.4%, a sensitivity of 45.10%, and a specificity of 96.04%.

In addition, the diagnostic function of SIRI, NLR, LMR, and SII, alone and in combination for AOS patients was assessed *via* the Receiver-operating characteristic (ROC) curves. It was revealed that the area under curve (AUC) was 0.701 [(0.632 - 0.763), *p <* 0.001] for SIRI, 0.734 [(0.668 - 0.793), *p <* 0.001] for NLR, 0.715 [(0.648 - 0.776), *p <* 0.001] for LMR, 0.730 [(0.663 - 0.790), *p <* 0.001] for SII, and 0.764 [(0.699 - 0.820), *p <* 0.001] for combined model. The optimal cut-off value with sensitivity and specificity for LMR was 5.41, 66.67%, and 67.33%; for NLR was 2.76, 41.18%, and 99.01%; for SII was 663.12, 49.02%, and 93.07%; for SIRI was 0.89, 52.94% and 78.22%, respectively. Whereas the combined model sensitivity and specificity were 45.10% and 96.04%, respectively (**Table 4**, **Figure 1**).

**Table 4:** Receiver-operating characteristic (ROC) curve of LMR, NLR, SII and SIRI for differentiating patients with AOS from Healthy Controls.

Variables	AUC	95%CI	Cut-off	sensitivity	specificity	*p*
**LMR**	0.715	0.648-0.776	5.41	66.67	67.33	<0.001^*^
**NLR**	0.734	0.668-0.793	2.76	41.18	99.01	<0.001^*^
**SII**	0.730	0.663-0.790	663.12	49.02	93.07	<0.001^*^
**SIRI**	0.701	0.632-0.763	0.89	52.94	78.22	<0.001^*^
**Combined**	0.764	0.699-0.820	–	45.10	96.04	<0.001^*^

LMR, Lymphocyte-monocyte ratio; NLR, Neutrophil–lymphocyte ratio; SII, systemic immune-inflammation index; SIRI, system inflammation response index; Combined, Joint LMR, NLR,SII and SIRI; *indicates statistical significance.

Based on multifactor regression and ROC analysis, correlation analysis was employed to assess the correlations between inflammatory indicators (LMR, PLR, SII, and SIRI) and PANSS scores. It was revealed that LMR was negatively correlation with the total PANSS score (*r =* -0.313, *p =* 0.001), negative factor score *(r =* -0.197, *p =* 0.047), and cognitive factor score *(r =* -0.266, *p =* 0.007). Furthermore, a positive correlation of NLR (*r =* 0.307, *p =* 0.002) was observed with the PANSS cognitive factor score. In addition, SII was negatively correlated with the PANSS positive factor score (*r = -*0.242, *p* = 0.014); while positively correlated with the negative factor score (*r =* 0.317, *p* = 0.001), and cognitive factor score (*r =* 0.250, *p* = 0.011). Lastly, SIRI had a negative correlation with the PANSS total score(*r* = 0.280, *p* = 0.004), and cognitive factor score (*r =* 0.363, *p <* 0.001). Detailed information regarding these correlations is presented in **Table 5**.

**Table 5:** Correlations between PNASS scores with LMR, PLR, PLR and SII.

Variables	PNASS-TS		PANSS-PFS		PANSS-NFS		PANSS-CFS		PANSS-EFS		PANSS-DFS	
	*r*	*p*	*r*	*p*	*r*	*p*	*r*	*p*	*r*	*p*	*r*	*p*
**LMR**	-0.313	0.001^*^	-0.077	0.440	-0.197	0.047^*^	-0.266	0.007^*^	-0.118	0.238	0.139	0.164
**NLR**	0.190	0.056	-0.149	0.135	0.135	0.176	0.307	0.002^*^	0.104	0.297	-0.097	0.333
**SII**	0.176	0.077	-0.242	0.014^*^	0.317	0.001^*^	0.250	0.011^*^	-0.050	0.618	-0.047	0.638
**SIRI**	0.280	0.004^*^	-0.031	0.756	0.157	0.115	0.363	<0.001^*^	0.188	0.058	-0.107	0.283

LMR, Lymphocyte-monocyte ratio; NLR, Neutrophil–lymphocyte ratio; SII, systemic immune-inflammation index; SIRI, system inflammation response index; PANSS, Positive and Negative Syndrome Scale; PNASS Total Score, PNASS-TS; PANSS-PFS, Positive Factor Score; PANSS-NFS, Negative Factor Score; PANSS-CFS, Cognitive Factor Score; PANSS-EFS, Excited Factor Score; PANSS-DFS, Depress Factor Score; *indicates statistical significance.

## Discussion

This research revealed that (1) compared with the healthy control cohort, the first-episode non-medicated Han Chinese AOS patients have markedly elevated levels of WBC count, LMR, neutrophil count, NLR, SIRI, PLR, and SII, while the lymphocyte count was notably decreased (2), binary multivariate stepwise analysis and ROC curve showed that LMR, NLR, SII, and SIRI could independently distinguish AOS patients and healthy adolescents (3), the correlation assessment revealed a negative correlation of LMR with the total PANSS, negative, and cognitive factor scores, a positive correlation of NLR with the cognitive factor score of PANSS, a negative correlation of SII with the positive factor score while a positive one with the negative and cognitive factor scores of PANSS, and positive correlation of SIRI with the total PANSS, and cognitive factor scores.

Currently, the pathogenic mechanism of schizophrenia is unknown. However, the immune and inflammation theory hypothesis has gained consensus in the academic community. Here, it was revealed that the indicators such as the WBC and neutrophil counts of AOS patients were increased, while the lymphocyte count decreased, which may reflect the abnormality of the patient’s immune system. This change in the immune system may be related to schizophrenia’s pathogenic mechanisms, such as the inflammatory response or the autoimmune process. WBCs are the key component of the immune system and are divided into multiple subtypes such as neutrophils, lymphocytes, and monocytes, *etc.*, which coordinate with each other to fight against infections, identify and remove foreign substances, and produce antibodies. Furthermore, WBCs are also a clinical marker for inflammation (51), Moody et al. found significantly higher total counts of WBCs, neutrophils, and monocytes in first-episode adult schizophrenia patients than in the healthy cohort; however, lymphocytes indicated no statistical difference (34). Dr. Jackson and Miller conducted a meta-analysis of 24 studies on the total number and classification counts of WBCs in schizophrenia patients and showed (31) that compared with the healthy individuals, the schizophrenia patients had markedly increased total WBCs, neutrophils, and monocyte counts. Furthermore, they also revealed that in the first episode of psychosis patients only indicated increased neutrophils and monocyte count but the total WBCs and lymphocyte count had no statistical difference compared with the healthy controls. These results were not affected by gender, BMI, age, and smoking. However, the data of the above research was consistent with the results of the present study, which might be because of the differences in inflammatory cytokine between adolescent and adult patients, or the differences in the included samples. Therefore, to comprehensively explain the results, further research is required. Although there are statistical differences in the number of WBCs and their subtypes between both cohorts, in most cases, the count alterations are mostly within the normal range for an individual patient. Therefore, it is currently difficult to distinguish AOS patients from healthy adolescents, which severely limits the application of WBCs and their subtypes in clinical practice, further indicating the requirement for novel inflammatory markers with good distinguishability.

This problem might be resolved by blood cell ratios. Recently, LMR, NLR, PLR, SII, and SIRI were identified as inflammatory indicators and studies have indicated their association with schizophrenia. The most studied of these indices include LMR, NLR, and PLR (31–34, 52, 53), while the researches on SII and SIRI are merely case reports (35, 36). Furthermore, the research objects are all adults and the results are not consistent. The present study showed that compared with the healthy controls, the drug-naive first-episode AOS patients had increased NLR and PLR, while decreased LMR, consistent with some previous studies. These studies aimed to evaluate adult schizophrenia (34, 54–56), including first-episode schizophrenia, and revealed that both adult and adolescent schizophrenia patients had similar immune-inflammatory responses and were not affected by antipsychotic drugs. SII and SIRI are relatively novel immune-inflammatory markers, which can more comprehensively reflect the inflammatory state of the body and immune balance. Compared with the LMR, NLR, and PLR, SII and SIRI are combinations of three inflammatory cells. Their increased levels are linked with various pathological conditions, the inflammatory degree of the disease, the curative effect, and the prognosis (29, 30, 57). This is the first study to employ two indicators for assessing first-episode AOS patients, and the results showed substantially increased levels of these markers compared with the healthy controls, consistent with studies on adult schizophrenia patients (35, 52, 58), validating the incidence of inflammatory response in schizophrenia patients. The inflammatory response might occur because of allergic diseases, infection, or autoimmune processes. Although the causal association between inflammation and schizophrenia remains unclear, the presence of an inflammatory state is confirmed.

Multivariate stepwise regression and ROC curve analysis showed that LMR, NLR, SII, and SIRI are independent predictive indicators for distinguishing AOS patients from their healthy counterparts, suggesting that these indicators may have an early diagnosis or prediction value for schizophrenia. Furthermore, it was revealed that PLR was not an independent predictive indicator of AOS schizophrenia. This might be because of the small sample size or the presence of intermediate factors between PLR and schizophrenia diagnosis, which further excluded PLR in the regression analysis. Subsequent research is warranted for further verification.

Although inflammatory markers including MLR, PLR, and NLR, may have potential diagnostic value in distinguishing schizophrenia patients from healthy individuals, their specificity for schizophrenia is low as they are also elevated in other mental disorders (22, 59). This cross-sectional study revealed that the regression model had a good performance, LMR, NLR, SII, and SIRI alone as well as the combined model could effectively distinguish between first-episode AOS patents and healthy controls. Therefore, LMR, NLR, SII, and SIRI can serve as potential biomarkers for the diagnosis of AOS, but this requires careful consideration, because the precise diagnostic markers for schizophrenia are complex, given its heterogeneous nature and the challenges associated with individual variability in treatment response. For a better diagnosis and comprehensive judgment, the precise schizophrenia diagnosis should be combined with the patient’s clinical symptoms, family history, and other auxiliary examinations or biological results, which are more scientific and reasonable.

The multi-factor regression and ROC analysis were carried out to further explore the relationships of LMR, NLR, SII, and SIRI with AOS-related clinical symptoms. Such a research design mitigates potential confounding factors present in single-factor analysis and assesses the diagnostic and predictive value of these independently related inflammatory markers. Furthermore, it provides more robust evidence for AOS patient’s diagnosis and treatment strategies as well as disease pathogenesis and clinical applications of the markers. This study revealed a negative association of LMR, while positively associated NLR, SII, and SIRI with the PANSS cognitive scores. This further validates that cognitive symptoms are a core component of schizophrenia (60). Studies have shown that the earlier onset of schizophrenia in patients is associated with more severe cognitive impairment (61). Cognitive dysfunction is also an important contributor to disability and impedes social functioning recovery in these individuals (60). Moreover, there is a lack of effective intervention and treatment strategies for this issue currently (60, 62). Our findings provide new insights for the treatment (especially in adolescents with cognitive symptoms), but it remains to be further investigations.

Several studies have investigated the role of adjunctive application of anti-inflammatory drugs for treating schizophrenia (2, 18) and the most researched of them include NSAIDs, such as aspirin and celecoxib, as well as immunosuppressants, like prednisolone and rituximab, *etc.*, especially for the treatment of first-episode psychosis and early schizophrenia (2, 18, 63). These data provide evidence for the importance of monitoring and treatment of abnormal immune-inflammatory conditions, further validating the results of this study. However, the specific clinical application of inflammatory indices still requires in-depth understanding and verification by more studies and clinical trials (2).

Certainly, this research has certain limitations. 1) Although all the factors that can affect the inflammatory state were strictly controlled, certain factors are still difficult to control, such as exercise condition, sleep pattern, and menstrual cycle. 2) Other immune function indicators, such as CRP and interleukin, were not evaluated. Furthermore, this is a single-center study and participants were selected from a single hospital the region was the same for convenient sampling, which may cause selection bias and reduce sample representativeness. 3) This is a retrospective cross-sectional research, therefore, the causal relationship between inflammatory indicators and AOS could not be assessed.

## Conclusion

In summary, it was revealed that the peripheral blood inflammation markers LMR, NLR, SII, and SIRI are independently associated with AOS and its clinical symptoms (especially the cognitive symptoms). Furthermore, these indices are potential markers of inflammatory status and AOS symptoms severity. Moreover, it was revealed that inflammation and schizophrenia pathogenesis were closely related. Therefore, this research provided new tools for the diagnosis, treatment, and assessment of AOS severity, and evidence for the anti-inflammatory treatment of schizophrenia, especially AOS.

## Data Availability

The raw data supporting the conclusions of this article will be made available by the authors, without undue reservation.

## Glossary

NLR: Neutrophil-lymphocyte ratio
LMR: Lymphocyte-Monocyte ratio
PLR: Platelet-lymphocyte ratio
SII: systemic immune-inflammation index
SIRI: system inflammation response index
AOS: Adolescent-onset Schizophrenia
IL-1/2/6/8: Interleukin-1/2/6/8
TNF-α: Tumor necrosis factor α
FEP: first-episode psychosis
CRP: C-reactive protein
NSAIDs: Nonsteroidal anti-inflammatory drugs
ICD-10: The 10^th^ edition of the International Classification of Diseases
BMI: Body mass index
MPV: Mean platelet volume
WBC: White blood cell count
OR: Odds ratios
CI: Confidence interval
ROC: Receiver operating characteristic
PANSS: Positive and Negative Syndrome Scale
PANSS-PF: Positive Factor
PANSS-NF: Negative Factor
PANSS-CF: Cognitive Factor
PANSS-EF: Excited Factor
PANSS-DFS: Depress Factor.
